# Genetic Diversity of SARS-CoV-2 among Travelers Arriving in Hong Kong

**DOI:** 10.3201/eid2710.211028

**Published:** 2021-10

**Authors:** Haogao Gu, Daniel K.W. Chu, Lydia D.J. Chang, Sammi S.Y. Cheuk, Shreya Gurung, Pavithra Krishnan, Daisy Y.M. Ng, Gigi Y.Z. Liu, Carrie K.C. Wan, Ruopeng Xie, Samuel S.M. Cheng, Benjamin J. Cowling, Dominic N.C. Tsang, Malik Peiris, Vijaykrishna Dhanasekaran, Leo L.M. Poon

**Affiliations:** School of Public Health, University of Hong Kong, Hong Kong, China (H. Gu, D.K.W. Chu, L.D.J. Chang, S.S.Y. Cheuk, S. Gurung, P. Krishnan, D.Y.M. Ng, G.Y.Z. Liu, C.K.C. Wan, R. Xie, S.S.M. Cheng, B.J. Cowling, M. Peiris, V. Dhanasekaran, L.L.M. Poon);; Department of Health Centre for Health Protection, Government of Hong Kong Special Administrative Region, Hong Kong (D.N.C. Tsang);; HKU-Pasteur Research Pole, University of Hong Kong, Hong Kong (M. Peiris, V. Dhanasekaran, L.L.M. Poon)

**Keywords:** coronavirus disease, COVID-19, disease transmission control, genomic diversity, public health, public health readiness, respiratory infections, reverse transcription PCR, SARS-COV-2, severe acute respiratory syndrome coronavirus 2, travel-related disease transmission, viruses, zoonoses

## Abstract

We sequenced 10% of imported severe acute respiratory syndrome coronavirus 2 infections detected in travelers to Hong Kong and revealed the genomic diversity of regions of origin, including lineages not previously reported from those countries. Our results suggest that international or regional travel hubs might be useful surveillance sites to monitor sequence diversity.

Hong Kong uses an elimination strategy to control coronavirus disease (COVID-19) that includes stringent travel restrictions to reduce the risk of introducing severe acute respiratory syndrome coronavirus 2 (SARS-CoV-2) into local communities (*1*). COVID-19 testing was mandated on departure and arrival for all inbound travelers. Compulsory 14-day home quarantine was put in place for all arrivals beginning March 19, 2020. Nonresidents were banned from entry after March 25. In subsequent months, persons arriving from high-risk locations were required to quarantine in hotels; by November, all arrivals had to quarantine in hotels. On December 25, the quarantine period was extended to 21 days. Predeparture COVID-19 testing was mandated for travelers inbound from high-risk locations. Furthermore, daily health declarations were required from all quarantined travelers and respiratory samples were collected on arrival, day 12, and day 19 (for 21-day quarantine) for reverse transcription PCR (RT-PCR) testing. As of April 25, 2021, authorities had recorded 11,731 RT-PCR–positive COVID-19 cases in Hong Kong. About 20% (2,350) of the laboratory-confirmed COVID-19 cases were considered imported, detected in persons thought to have been infected outside of Hong Kong. Here, we report the analyses of 10% of these imported cases through genome sequencing. 

## The Study

A total of 2,192 COVID-19–positive travelers arrived in Hong Kong during January 2020–March 2021 (Appendix 1 Figure 1). Stratifying cases by departure location (Appendix 1 Table 1) showed that 10 countries accounted for 77.8% of all imported cases during this period: United Kingdom (406), Philippines (318), India (309), Pakistan (245), Indonesia (149), United States (131), Nepal (75), Russia (40), France (33), and United Arab Emirates (25). After compulsory COVID-19 RT-PCR screening on arrival at the airport began on April 7, 2020, authorities detected 1,102 cases; 80% (886) of case-patients were asymptomatic at the time of testing. Of 491 case-patients testing SARS-CoV-2–positive during quarantine, 69% were asymptomatic and cases were detected a mean (+ SD) of 11.3 +4.32 days after arrival. This finding indicates that many COVID-19 cases from quarantined travelers were only identified during the first compulsory testing on day 12. These findings support Hong Kong’s stringent follow-up measures for inbound travelers to prevent introduction of SARS-CoV-2 into communities. 

**Table 1:** Severe acute respiratory syndrome coronavirus 2 variants of concern and variants of interest identified in imported cases in Hong Kong, January 2020–March 2021

Pango lineage	Total cases	Country (no. cases)
B.1.1.7*	39	Pakistan (13), Philippines (8), United Kingdom (7), United Arab Emirates (3), India (2), Netherlands (2), Canada (1), Ireland (1), South Korea (1), Switzerland (1)
B.1.351*	7	Philippines (5), Bangladesh (1), United Kingdom/South Africa (1)
B.1.526†	1	United States (1)
B.1.617†	1	India (1)
P.3†	6	Philippines (6)

*Variant of concern. †Variant of interest.

To estimate the viral sequence diversity among these imported cases, we performed next-generation sequencing on 10% (221) of clinical samples collected (*2*,*3*) (Appendix). We selected a greater proportion of samples (204) beginning in June 2020 when greater genetic diversity began to appear globally. The number of samples we sequenced by country of origin was proportional to all cases detected in travelers from that country (R = 0.91). 

Using the Pangolin classification system (https://github.com/hCoV-2019/pangolin), we detected 58 different SARS-CoV-2 lineages; the most common were B.1.1.7 (39), B.1.1.63 (21), B.1.36 (18), B.1 (17), and B.1.1 (17) (Figure; Appendix 1 Table 2). We detected 2 variants of concern (VOC) and 3 variants of interest (VOI; Table 1) (*4**,**5*). VOC B.1.1.7 (Alpha variant), which began spreading rapidly in the United Kingdom in November 2020 (*6*,*7*), was the most common VOC (39) in our study. We first detected this lineage in a passenger arriving from the United Kingdom on December 13, 2020, and we subsequently detected it in another 38 travelers from other countries, predominantly from the Philippines and Pakistan (Table 1). This finding corresponds with data from global surveillance that indicate this lineage has been circulating over a wide geographic range beginning in December 2020. The second VOC, B.1.351 (Beta), which was first reported to circulate widely in South Africa beginning in November 2020 (*8*), we first detected on December 16 in an arriving passenger with a recent travel history in the United Kingdom and South Africa (*1*). Subsequent cases caused by this variant were detected only in March 2021 in travelers from the Philippines (*5*) and Bangladesh (*1*). All 3 of the VOI we detected were imported from the countries where they were first reported to have emerged: B.1.526 (Iota) from the United States, B.1.617 (Kappa) from India, and P.3 (Theta) from the Philippines (M.K. Annavajhala et al., unpub data, https://doi.org/10.1101/2021.02.23.21252259; S. Cherian et al., unpub data, https://doi.org/10.1101/2021.04.22.440932; F.A. Tablizo et al., unpub data, https://doi.org/10.1101/2021.03.03.21252812). Based on sequences detected in samples from case-patients, B.1.526 was imported on March 20, B.1.617 on March 25, and P.3 on January 21, 2021. These variants were first reported to spread rapidly in these countries during February (B1.526 and B.1.617) and March 2021 (P.3), indicating that testing arrivals from outside of Hong Kong and sequencing positive samples might enable us to capture information about variants circulating in other geographic locations. 

**Figure:**
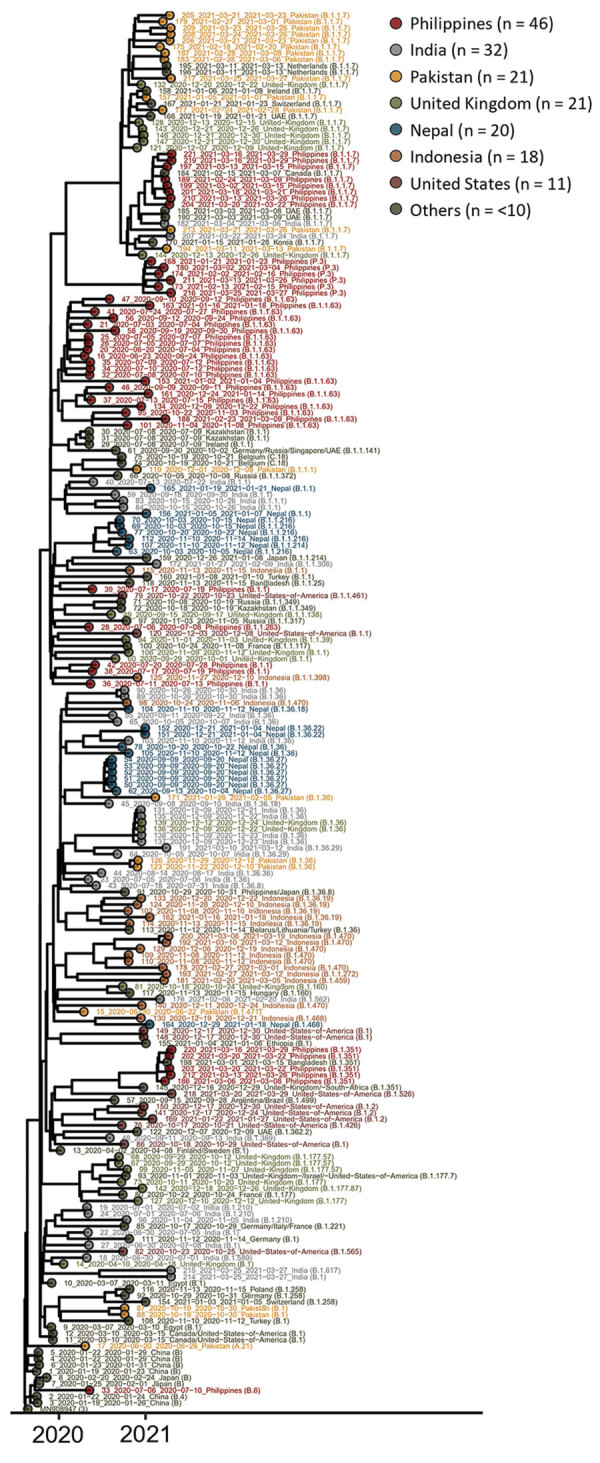
Maximum clade credibility phylogenetic tree of sequences of severe acute respiratory syndrome coronavirus 2 imported to Hong Kong, January 2020–March 2021 (Appendix 1). Date, country, and lineage information are provided.

Fifty percent of our cases were imported from 5 middle-income countries in Asia: India, Indonesia, Nepal, Pakistan, and the Philippines (https://databank.worldbank.org/data/download/site-content/CLASS.xls; Appendix Table 1). We wanted to compare the genomic diversity of SARS-CoV-2 imported from these countries with those reported in the GISAID database (https://www.gisaid.org). However, the Philippines, Nepal, and Pakistan had limited SARS-CoV-2 sequence information in the GISAID database (Table 2) (*9*). Of the 3 VOC or VOI we identified in travelers from the Philippines (Table 2), B.1.351 was not among sequences the Philippines submitted to GISAID, but the March 6–20, 2021, arrival dates of the 5 case-patients with B.1.351 suggest unreported domestic circulation of that lineage. Similarly, Nepal had reported to GISAID only 15 of the 20 viral sequences from 8 lineages we had identified. Other countries also had not previously reported several lineages we identified to GISAID, including 3 from India and 1 each from Pakistan and Indonesia. We did not analyze samples from travelers from some countries, either because they had their own extensive domestic sequencing efforts or we had few samples from these countries (<5 per country).

**Table 2:** Severe acute respiratory syndrome coronavirus 2 lineages imported from different countries in Asia into Hong Kong, January 2020–March 2021

Country	No. sequences from GISAID*	**No. sequences in this study**	**Lineages found in this study**
India	11,435	32	B.1, B.1.1, B.1.1.1, B.1.1.306, B.1.1.7, B.1.210, B.1.36, B.1.36.18, B.1.36.29, B.1.36.36,† B.1.36.8, B.1.369, B.1.562,† B.1.589,† B.1.617
Indonesia	1,170	18	B.1.1, B.1.1.272,† B.1.1.398, B.1.36.19, B.1.459, B.1.468, B.1.470
Philippines	188	47	B.1.1, B.1.1.263, B.1.1.63, B.1.1.7, B.1.351,† B.6, P.3
Pakistan	136	21	A.21,† B.1, B.1.1.1, B.1.1.7, B.1.36, B.1.471
Nepal	15	20	B.1.1, B.1.1.214,† B.1.1.216, B.1.36, B.1.36.18,† B.1.36.22,† B.1.36.27,† B.1.468†

*GISAID, https://www.gisaid.org.

†Lineage not reported by the corresponding country.

We further compared GISAID data with our data from the Philippines, Nepal, and Pakistan. We retrieved the earliest collection date for each lineage we detected that these countries had also reported to GISAID; some of those dates were close to the first dates of arrival for case-patients with those lineages in our study. In fact, in over half of those lineages reported in both sources, we identified the lineage either before or <1 month after it was reported by the country (Appendix Table 3), highlighting the potential use of this method of surveillance to assess genomic diversity in regions with limited sequence information. 

The emergence of VOC and VOI in different geographic locations highlights the need for global-level genomic surveillance of SARS-CoV-2 (*10*), but genomic sequencing information from some regions remains incomplete. Our findings suggest that travel hubs such as Hong Kong can be used as surveillance sites to identify infected travelers from regions with widespread circulation of lineages of interest. Such indirect surveillance might provide useful data to partially reveal virus diversity in countries with limited sequence information, leading to better preparedness for and response to newly emerging SARS-CoV-2 variants. However, findings from these indirect analyses are likely to be only partial and skewed by the level of passenger traffic to destination countries from various points of departure. Also, the extent of different virus lineages circulating in a country of departure may have affected our observations; lineages that circulate at a low level in a country of interest might be missed by our current strategy. Optimizing this approach, such as by directing sequencing efforts toward travelers departing from targeted countries or regions rather than at the points of arrival, might help overcome those limitations. 

Appendix 1 Additional methods and results from study of SARS-CoV-2 among travelers arriving in Hong Kong.

Appendix 2 Acknowledgments of sequences from GISAID used for study of SARS-CoV-2 among travelers arriving in Hong Kong.
