# Supplementary material for: Genetic Diversity of SARS-CoV-2 among Travelers Arriving in Hong Kong
Source: Emerg Infect Dis. 2021 Oct;27(10):2666–8. doi: 10.3201/eid2710.211028 (PMC8462320; doi:10.3201/eid2710.211028)
Supplement: Appendix 1 — Additional methods and results from study of SARS-CoV-2 among travelers arriving in Hong Kong. [file 21-1028-Techapp-s1.pdf]

Article DOI: https://doi.org/10.3201/eid2710.211028

Genetic Diversity of SARS-CoV-2 among Travelers Arriving in Hong Kong

Appendix

Additional Methods

Sequencing

Followed a recommendation from the European Centre for Disease Prevention and Control, we selected 10% of our imported cases for sequence analysis (https://ec.europa.eu/info/sites/default/files/communication-united-front-beat-covid-19_en.pdf). We selected clinical samples with a cycle threshold value <30 for the next generation sequencing study. Beginning in June 2020, we sequenced (mean ±SD) 20.7 ±10.7 samples from each month. We sent RNA samples of interest to a World Health Organization reference laboratory at the University of Hong Kong for full genome analyses (IRB no. UW 20–168). We deduced near full-length genomes from all available samples (sequence length >29,700 nt; sequence coverage >100) using a Illumina sequencing protocol described elsewhere (*1*,*2*). Briefly, we performed reverse transcription PCR on the virus genome with multiple gene-specific primers targeting different regions. We then subjected the synthesized cDNA to multiple overlapping 2 kb PCRs for full-genome amplification. We pooled PCR amplicons obtained from the same specimen and sequenced them using Nova sequencing platform (Illumina; https://www.illumina.com). We prepared the sequencing library by Nextera XT (Illumina).

Phylogenetic analysis

We mapped generated sequencing reads to a reference virus genome using Burrows-Wheeler Aligner (http://bio-bwa.sourceforge.net), and generated genome consensus using Geneious version 11.1.4 (https://www.geneious.com). With the full set of sequences imported to Hong Kong in this study, we used the BEAST v1.10.4 GMRF Bayesian Skyride (http://www.beast2.org/features/tree-prior-gmrf-bayesian-skyride.html) coalescent model (*3*). We used the HKY+Γ nucleotide substitution model along with an uncorrelated relaxed molecular

clock with the lognormal distribution. We ran the MCC-seq (methylC-capture sequencing) chains 400 million times, sampling every 40,000 and discarding 10% as burn-in. Sufficient sampling was inspected by Tracer v1.7.1 (https://github.com/beast-dev/tracer/releases/tag/v1.7.2) (*4*), confirming that the effective sample size (ESS) of each parameter was >1000. We classified lineages using PANGO nomenclature proposal (pangoLEARN v. 2021–04–01; https://github.com/cov-lineages/pangoLEARN) (*5*).

References

1. Choi EM, Chu DKW, Cheng PKC, Tsang DNC, Peiris M, Bausch DG, et al. In-flight transmission of SARS-CoV-2. Emerg Infect Dis. 2020;26:2713–6. PubMed https://doi.org/10.3201/eid2611.203254

2. Sit THC, Brackman CJ, Ip SM, Tam KWS, Law PYT, To EMW, et al. Infection of dogs with SARS-CoV-2. Nature. 2020;586:776–8. PubMed https://doi.org/10.1038/s41586-020-2334-5

3. Suchard MA, Lemey P, Baele G, Ayres DL, Drummond AJ, Rambaut A. Bayesian phylogenetic and phylodynamic data integration using BEAST 1.10. Virus Evol. 2018;4:vey016. PubMed https://doi.org/10.1093/ve/vey016

4. Rambaut A, Drummond AJ, Xie D, Baele G, Suchard MA. Posterior summarization in Bayesian phylogenetics using Tracer 1.7. Syst Biol. 2018;67:901–4. PubMed https://doi.org/10.1093/sysbio/syy032

5. Rambaut A, Holmes EC, O'Toole Á, Hill V, McCrone JT, Ruis C, et al. A dynamic nomenclature proposal for SARS-CoV-2 lineages to assist genomic epidemiology. Nat Microbiol. 2020;5:1403–7. PubMed https://doi.org/10.1038/s41564-020-0770-5

Appendix Table 1. Country of origin of imported COVID-19 cases*

Country	No. total cases	No. sequenced cases
UK	406	21
Philippines	318	46
India	309	32
Pakistan	245	21
Indonesia	149	18
USA	131	11
Nepal	75	20
Russia	40	3
France	33	2
UAE	25	4
Japan	23	3
Turkey	21	2
Canada	20	1
Germany	19	2
Switzerland	17	2
Kazakhstan	15	3
Mainland China	14	6
Bangladesh	13	2
Egypt	12	2
Ethiopia	12	1
Spain	12	0
Ukraine	12	0
Bolivia/Canada/Peru	8	0
Singapore/Malaysia/China	8	0
Belgium	7	2
Brazil	7	0
Canada/USA	7	2
Morocco	7	0
Netherlands	7	2
Unknown	7	0
Australia	5	0
Ecuador	5	0
Finland/Singapore/Sweden	5	0
France/Switzerland	5	0
Ireland	5	2
UK/USA	5	0
Poland	4	1
Romania	4	0
Argentina/Brazil/Chile/Peru	3	0
Denmark	3	0
France/Spain	3	0
Malaysia	3	0
Peru	3	0
Serbia	3	0
South Africa	3	0
Sweden	3	0
Switzerland/UK	3	0
Argentina	2	0
Australia/UAE/UK	2	0
Austria/France	2	0
Austria/Switzerland	2	0
Bahrain	2	0
Belarus	2	0
Belarus/Lithuania/Turkey	2	1
Czech/France/UK	2	0
Finland/Iceland	2	0
France/Spain/UK	2	0
France/UK	2	0
Hungary	2	1
Iceland/UK	2	0
Iran	2	0
Japan/Thailand	2	0

Country	No. total cases	No. sequenced cases
Korea	2	1
Mexico	2	0
Myanmar	2	0
Netherlands/UK	2	0
Nigeria	2	0
Portugal/Switzerland/USA	2	0
Qatar	2	0
Togo	2	0
Tunisia	2	0
Africa/Brazil	1	0
Albania	1	0
Albania/Turkey/UAE	1	0
Argentina/Brazil	1	1
Argentina/Brazil/Chile	1	0
Argentina/Chile	1	0
Australia/France	1	0
Austria	1	0
Austria/Germany	1	0
Austria/Netherlands/Switzerland	1	0
Austria/Spain/UK/Thailand	1	0
Azerbaijan	1	0
Bahrain/USA/Belgium/Luxembourg	1	0
Belgium/Ethiopia	1	0
Belgium/China/Ethiopia	1	0
Botswana	1	0
Brazil/UK	1	0
Bulgaria	1	0
Bulgaria/Turkey	1	0
Canada/India	1	0
Canada/UK	1	0
Canada/UK/USA	1	0
Czech Republic	1	0
Denmark/Ireland/Poland/UK/Sweden	1	0
Egypt/UAE	1	0
Ethiopia/UAE/Belgium/Norway/Korea	1	0
Ethiopia/Belgium	1	0
Ethiopia/Belgium/Norway/Korea	1	0
Ethiopia/Spain	1	0
Finland/Sweden	1	1
France/Austria	1	0
France/Morocco	1	0
France/Netherlands	1	0
France/Netherlands/UK	1	0
France/Switzerland/UK	1	0
France/Taiwan	1	0
France/UK/USA	1	0
Gambia	1	0
Germany/Italy/France	1	1
Germany/Italy/UAE	1	0
Germany/Japan	1	0
Germany/Netherlands/Switzerland	1	0
Germany/Russia/Singapore/UAE	1	1
Germany/UK	1	0
Ghana	1	0
India/Qatar	1	0
India/Netherlands/UK	1	0
India/UK	1	0
Indonesia/Switzerland	1	0
Indonesia/UK	1	0
Ireland/Spain/Malta/UK	1	0
Italy	1	0
Italy/Malta/Ukraine	1	0
Japan/Korea	1	0

Country	No. total cases	No. sequenced cases
Kazakhstan/Turkey	1	0
Kenya	1	0
Luxemburg/USA/Azerbaijan/Japan	1	0
Maldives/Qatar	1	0
Malta	1	0
Mauritania	1	0
Mexico/Brazil	1	0
Mexico/UK/USA	1	0
Montenegro	1	0
Netherlands/UK	1	0
Netherlands/Thailand/UK	1	0
Norway	1	0
Philippines/Japan	1	1
Philippines/USA	1	0
Portugal/Spain	1	0
Portugal/Spain/UK	1	0
Russia/South Africa	1	0
Russia/Turkey/Netherlands/Thailand	1	0
Rwanda	1	0
Saudi Arabia	1	0
Singapore	1	0
Singapore/Malaysia/Philippines	1	0
Singapore/UK	1	0
South Africa/UK	1	0
Spain/UK	1	0
Sri Lanka/UK	1	0
Switzerland/Germany	1	0
Switzerland/Sweden	1	0
Syria	1	0
Tanzania	1	0
Turkey/China/USA/Netherlands	1	0
Turkey/Russia	1	0
Ukraine/Czech/Korea	1	0
UK/Israel/USA	1	1
UK/Jamaica	1	0
UK/South Africa	1	1
UK/UAE	1	0
UK/USA/Barbados	1	0
UK/Germany/UAE	1	0
UK/Indonesia	1	0
UK/Spain	1	0
UK/Switzerland	1	0
UK/UAE/India	1	0
USA/Australia	1	0
USA/Japan/Korea	1	0
USA/France/Japan	1	0
Total	2,192	221

*Case-patients with a recent travel history to multiple countries before arriving in Hong Kong analyzed separately.

Appendix Table 2. Pango lineages identified in this study

Pango lineage	No. cases
A.21	1
B	7
B.1	17
B.1.1	17
B.1.1.1	2
B.1.1.117	1
B.1.1.138	1
B.1.1.141	1
B.1.1.214	2
B.1.1.216	5
B.1.1.25	1
B.1.1.263	1
B.1.1.272	1
B.1.1.306	1
B.1.1.317	1
B.1.1.349	2
B.1.1.372	1
B.1.1.39	1
B.1.1.398	1
B.1.1.461	1
B.1.1.63	21
B.1.1.7	39
B.1.160	2
B.1.177	3
B.1.177.15	0
B.1.177.57	3
B.1.177.7	1
B.1.177.87	1
B.1.2	3
B.1.210	3
B.1.221	1
B.1.258	3
B.1.351	7
B.1.36	18
B.1.36.18	2
B.1.36.19	5
B.1.36.22	2
B.1.36.27	6
B.1.36.29	2
B.1.36.36	1
B.1.36.8	2
B.1.362.2	1
B.1.369	1
B.1.426	1
B.1.459	1
B.1.468	2
B.1.470	8
B.1.471	1
B.1.499	1
B.1.526	1
B.1.562	1
B.1.565	1
B.1.589	1
B.1.617	1
B.4	1
B.6	1
C.18	2
P.3	6

Appendix Table 3. The first sample collection date reported by country and the arrival date of the first imported cases detected in this study.

Country, Pango lineage	Date reported to GISAID by country	First imported case in this study
Philippines		
B.1.1†	2020 Jun 25	2020 Jul 11
B.1.1.263	2020 Oct 12	2020 Jul 6
B.1.1.63†	2020 Jun	2020 Jun 23
B.1.1.7	2020 Dec 10	2021 Feb 24
B.1.351*	No sequence	2021 Mar 6
B.6	Mar 4	2020 Jul 6
P.3[#]	2021 Jan 16	2021 Jan 21
Nepal		
B.1.1	2020 Aug 3	2021 Jan 5
B.1.1.214*	No sequence	2020 Nov 10
B.1.1.216	2020 Jul 30	2020 Oct 3
B.1.36	2020 Jul 30	2020 Oct 20
B.1.36.18*	No sequence	2020 Nov 10
B.1.36.22*	No sequence	2020 Dec 21
B.1.36.27*	No sequence	2020 Sep 9
B.1.468*	No sequence	2020 Dec 29
Pakistan		
A.21*	No sequence	2020 Jun 20
B.1	2020 May 3	2020 Oct 19
B.1.1.1	2020 Jul 7	2020 Dec 1
B.1.1.7†	2020 Dec 25	2021 Jan 5
B.1.36	2020 Jun 2	2020 Nov 22
B.1.471†	2020 May 20	2020 Jun 20

*Lineage not reported by country
†Lineage with the sample collection date reported by the country close to our first imported case

Appendix Table 4. COVID-19 sequences reported in this study

Virus name	GISAID no.
hCoV-19/Hong Kong/VM20127572–1/2020	EPI_ISL_1718252
hCoV-19/Hong Kong/CM21000014/2021	EPI_ISL_1718270
hCoV-19/Hong Kong/VM20030723–1/2020	EPI_ISL_1718271
hCoV-19/Hong Kong/VM20035559/2020	EPI_ISL_1731057
hCoV-19/Hong Kong/VM20028080/2020	EPI_ISL_1749650
hCoV-19/Hong Kong/CM20000284/2020	EPI_ISL_1749657
hCoV-19/Hong Kong/VM20123555–1/2020	EPI_ISL_1749662
hCoV-19/Hong Kong/VM20089384/2020	EPI_ISL_1749669
hCoV-19/Hong Kong/VM21000356/2021	EPI_ISL_1761404
hCoV-19/Hong Kong/VM20194433/2020	EPI_ISL_1761417
hCoV-19/Hong Kong/VM20153752/2020	EPI_ISL_1761420
hCoV-19/Hong Kong/VM20127230/2020	EPI_ISL_1761429
hCoV-19/Hong Kong/VB20239988/2020	EPI_ISL_1761431
hCoV-19/Hong Kong/VM20030143/2020	EPI_ISL_1761447
hCoV-19/Hong Kong/VM20134624/2020	EPI_ISL_1793811
hCoV-19/Hong Kong/VM20030475/2020	EPI_ISL_1793822
hCoV-19/Hong Kong/CM20000427–1/2020	EPI_ISL_1793836
hCoV-19/Hong Kong/CM20000444–1/2020	EPI_ISL_1793841
hCoV-19/Hong Kong/CM20000283/2020	EPI_ISL_1811327
hCoV-19/Hong Kong/CM20000156/2020	EPI_ISL_1811334
hCoV-19/Hong Kong/VM20123558–1/2020	EPI_ISL_1811338
hCoV-19/Hong Kong/VM20124082/2020	EPI_ISL_1811349
hCoV-19/Hong Kong/VM20196332/2020	EPI_ISL_1811353
hCoV-19/Hong Kong/VM20143876/2020	EPI_ISL_1819089
hCoV-19/Hong Kong/VM20194110/2020	EPI_ISL_1819091
hCoV-19/Hong Kong/VM21000357/2021	EPI_ISL_1819095
hCoV-19/Hong Kong/VM20056300/2020	EPI_ISL_1819119
hCoV-19/Hong Kong/VM20193891/2020	EPI_ISL_1819121
hCoV-19/Hong Kong/VM20121505/2020	EPI_ISL_1819127
hCoV-19/Hong Kong/VM20197060/2020	EPI_ISL_1819131
hCoV-19/Hong Kong/VM20126621/2020	EPI_ISL_1819143
hCoV-19/Hong Kong/VM20146611/2020	EPI_ISL_1819149
hCoV-19/Hong Kong/VM20031164/2020	EPI_ISL_1819169
hCoV-19/Hong Kong/VM20123560–1/2020	EPI_ISL_1828743
hCoV-19/Hong Kong/VM20194436/2020	EPI_ISL_1828744
hCoV-19/Hong Kong/WHP1765/2020	EPI_ISL_1828746

Virus name	GISAID no.
hCoV-19/Hong Kong/VM20120084/2020	EPI_ISL_1828751
hCoV-19/Hong Kong/CM20000179/2020	EPI_ISL_1828754
hCoV-19/Hong Kong/VM20194108/2020	EPI_ISL_1828757
hCoV-19/Hong Kong/VM20149336/2020	EPI_ISL_1828761
hCoV-19/Hong Kong/VM20141728–1/2020	EPI_ISL_1828764
hCoV-19/Hong Kong/CM21000050/2021	EPI_ISL_1828774
hCoV-19/Hong Kong/CM21000044/2021	EPI_ISL_1897307
hCoV-19/Hong Kong/VB20239959/2020	EPI_ISL_1897350
hCoV-19/Hong Kong/VM20187651/2020	EPI_ISL_1914430
hCoV-19/Hong Kong/VM20128239/2020	EPI_ISL_1914438
hCoV-19/Hong Kong/VB20235197/2020	EPI_ISL_1914451
hCoV-19/Hong Kong/VM20131770/2020	EPI_ISL_1914464
hCoV-19/Hong Kong/VM20193653/2020	EPI_ISL_1914475
hCoV-19/Hong Kong/CM20000211/2020	EPI_ISL_1914476
hCoV-19/Hong Kong/VM20197057/2020	EPI_ISL_1914477
hCoV-19/Hong Kong/CM20000221–1/2020	EPI_ISL_1914478
hCoV-19/Hong Kong/VM20127500/2020	EPI_ISL_1914479
hCoV-19/Hong Kong/VM20030473/2020	EPI_ISL_1914480
hCoV-19/Hong Kong/VM20126594/2020	EPI_ISL_1914481
hCoV-19/Hong Kong/VM20109249/2020	EPI_ISL_1914482
hCoV-19/Hong Kong/VM20137973/2020	EPI_ISL_1914483
hCoV-19/Hong Kong/VM20193204/2020	EPI_ISL_1914484
hCoV-19/Hong Kong/CM21000031/2021	EPI_ISL_1914485
hCoV-19/Hong Kong/VM200–58490/2020	EPI_ISL_1914486
hCoV-19/Hong Kong/VB20227521/2020	EPI_ISL_1914487
hCoV-19/Hong Kong/VM20029491/2020	EPI_ISL_1914488
hCoV-19/Hong Kong/VB20299920/2020	EPI_ISL_1914489
hCoV-19/Hong Kong/VM20129417/2020	EPI_ISL_1914490
hCoV-19/Hong Kong/VM20154035/2020	EPI_ISL_1914491
hCoV-19/Hong Kong/VM20149166–1/2020	EPI_ISL_1914492
hCoV-19/Hong Kong/VM20139861/2020	EPI_ISL_1914493
hCoV-19/Hong Kong/VM20195199/2020	EPI_ISL_1914494
hCoV-19/Hong Kong/VM20136554/2020	EPI_ISL_1914495
hCoV-19/Hong Kong/VM20149334/2020	EPI_ISL_1914496
hCoV-19/Hong Kong/CM20000176/2020	EPI_ISL_1914497
hCoV-19/Hong Kong/CM20000461–1/2020	EPI_ISL_1914498
hCoV-19/Hong Kong/VM21000250/2021	EPI_ISL_1914499
hCoV-19/Hong Kong/VM20037354/2020	EPI_ISL_1914500
hCoV-19/Hong Kong/VM20027143–1/2020	EPI_ISL_1914501
hCoV-19/Hong Kong/VM20027699/2020	EPI_ISL_1914502
hCoV-19/Hong Kong/VM20194109/2020	EPI_ISL_1914503
hCoV-19/Hong Kong/VM20030364/2020	EPI_ISL_1914504
hCoV-19/Hong Kong/VM20082690/2020	EPI_ISL_1914505
hCoV-19/Hong Kong/VM20032529/2020	EPI_ISL_1914506
hCoV-19/Hong Kong/CH20186398/2020	EPI_ISL_1914507
hCoV-19/Hong Kong/VM20136998/2020	EPI_ISL_1914508
hCoV-19/Hong Kong/CM21000015/2021	EPI_ISL_1914509
hCoV-19/Hong Kong/VM20152069/2020	EPI_ISL_1914510
hCoV-19/Hong Kong/VM21001490/2021	EPI_ISL_1914511
hCoV-19/Hong Kong/CM20000193/2020	EPI_ISL_1914512
hCoV-19/Hong Kong/VM20063224/2020	EPI_ISL_1914513
hCoV-19/Hong Kong/VM20138739/2020	EPI_ISL_1914514
hCoV-19/Hong Kong/CV20089510/2020	EPI_ISL_1914515
hCoV-19/Hong Kong/VM20193892/2020	EPI_ISL_1914516
hCoV-19/Hong Kong/VB20329247/2020	EPI_ISL_1914517
hCoV-19/Hong Kong/VM20033948/2020	EPI_ISL_1914518
hCoV-19/Hong Kong/VM20195030/2020	EPI_ISL_1914519
hCoV-19/Hong Kong/VM20139199–1/2020	EPI_ISL_1914520
hCoV-19/Hong Kong/VM20030363/2020	EPI_ISL_1914521
hCoV-19/Hong Kong/CM20000424–1/2020	EPI_ISL_1914522
hCoV-19/Hong Kong/CM21000021/2021	EPI_ISL_1914523
hCoV-19/Hong Kong/VM20149375/2020	EPI_ISL_1914524
hCoV-19/Hong Kong/VM20196066/2020	EPI_ISL_1914525
hCoV-19/Hong Kong/VB20245514/2020	EPI_ISL_1914526
hCoV-19/Hong Kong/CM21000063/2021	EPI_ISL_1914527
hCoV-19/Hong Kong/VM20141448/2020	EPI_ISL_1914528
hCoV-19/Hong Kong/VM20197058/2020	EPI_ISL_1914529
hCoV-19/Hong Kong/VM20122595/2020	EPI_ISL_1914530
hCoV-19/Hong Kong/VM20126174/2020	EPI_ISL_1914531

Virus name	GISAID no.
hCoV-19/Hong Kong/CM21000012/2021	EPI_ISL_1914532
hCoV-19/Hong Kong/VM20134148/2020	EPI_ISL_1914533
hCoV-19/Hong Kong/VM20124689–1/2020	EPI_ISL_1914534
hCoV-19/Hong Kong/VB20227528/2020	EPI_ISL_1914535
hCoV-19/Hong Kong/VM20139862/2020	EPI_ISL_1914536
hCoV-19/Hong Kong/CM21000019/2021	EPI_ISL_1914537
hCoV-19/Hong Kong/VM20141727/2020	EPI_ISL_1914538
hCoV-19/Hong Kong/CM20000166/2020	EPI_ISL_1914539
hCoV-19/Hong Kong/VM20196067/2020	EPI_ISL_1914540
hCoV-19/Hong Kong/CV20083787/2020	EPI_ISL_1914541
hCoV-19/Hong Kong/VM20135297/2020	EPI_ISL_1914542
hCoV-19/Hong Kong/VM20132139/2020	EPI_ISL_1914543
hCoV-19/Hong Kong/VM20153747/2020	EPI_ISL_1914544
hCoV-19/Hong Kong/VM20080417/2020	EPI_ISL_1914545
hCoV-19/Hong Kong/VM20186034/2020	EPI_ISL_1914546
hCoV-19/Hong Kong/VM20031429/2020	EPI_ISL_1914547
hCoV-19/Hong Kong/CM21000055/2021	EPI_ISL_1914548
hCoV-19/Hong Kong/VM20120246/2020	EPI_ISL_1914549
hCoV-19/Hong Kong/VB20221937/2020	EPI_ISL_1914550
hCoV-19/Hong Kong/CM20000267/2020	EPI_ISL_1914551
hCoV-19/Hong Kong/CM21000045/2021	EPI_ISL_1914552
hCoV-19/Hong Kong/CM20000096/2020	EPI_ISL_1914553
hCoV-19/Hong Kong/CH20186399/2020	EPI_ISL_1914554
hCoV-19/Hong Kong/VM20030059/2020	EPI_ISL_1914555
hCoV-19/Hong Kong/VM20149376/2020	EPI_ISL_1914556
hCoV-19/Hong Kong/VM20128241/2020	EPI_ISL_1914557
hCoV-19/Hong Kong/VM20030724/2020	EPI_ISL_1914558
hCoV-19/Hong Kong/CM20000160/2020	EPI_ISL_1914559
hCoV-19/Hong Kong/VM20189132/2020	EPI_ISL_1914560
hCoV-19/Hong Kong/CV20080655/2020	EPI_ISL_1914561
hCoV-19/Hong Kong/VM20031430/2020	EPI_ISL_1914562
hCoV-19/Hong Kong/VB20304975/2020	EPI_ISL_1914563
hCoV-19/Hong Kong/VM20196646/2020	EPI_ISL_1914564
hCoV-19/Hong Kong/VM20140376/2020	EPI_ISL_1914565
hCoV-19/Hong Kong/VM20195031/2020	EPI_ISL_1914566
hCoV-19/Hong Kong/VM20121196/2020	EPI_ISL_1914567
hCoV-19/Hong Kong/VM20153748/2020	EPI_ISL_1914568
hCoV-19/Hong Kong/CV20080825/2020	EPI_ISL_1914569
hCoV-19/Hong Kong/VM20187650/2020	EPI_ISL_1914570
hCoV-19/Hong Kong/CM20000216/2020	EPI_ISL_1914571
hCoV-19/Hong Kong/VM20123554–1/2020	EPI_ISL_1914572
hCoV-19/Hong Kong/VM20123559–1/2020	EPI_ISL_1914573
hCoV-19/Hong Kong/CM21000064/2021	EPI_ISL_1914574
hCoV-19/Hong Kong/VM200–44231/2020	EPI_ISL_1914575
hCoV-19/Hong Kong/VM20137971/2020	EPI_ISL_1919948
hCoV-19/Hong Kong/VM20001794–1/2020	EPI_ISL_1963574
hCoV-19/Hong Kong/VB20019871–1/2020	EPI_ISL_1963575
hCoV-19/Hong Kong/VB20026565–1/2020	EPI_ISL_1963576
hCoV-19/Hong Kong/VM20001403–1/2020	EPI_ISL_1963577
hCoV-19/Hong Kong/VM20001776–1/2020	EPI_ISL_1963578
hCoV-19/Hong Kong/VM20002162–1/2020	EPI_ISL_1963579
hCoV-19/Hong Kong/VM20002849–1/2020	EPI_ISL_1963580
hCoV-19/Hong Kong/VB20017371/2020	EPI_ISL_1963581
hCoV-19/Hong Kong/WHP125/2020	EPI_ISL_1963582
hCoV-19/Hong Kong/WHP126/2020	EPI_ISL_1963583
hCoV-19/Hong Kong/WHP423/2020	EPI_ISL_1963584
hCoV-19/Hong Kong/WHP424/2020	EPI_ISL_1963585
hCoV-19/Hong Kong/WHP869/2020	EPI_ISL_1963586
hCoV-19/Hong Kong/VM20010001/2020	EPI_ISL_1963587
hCoV-19/Hong Kong/VM20033958/2020	EPI_ISL_1963588
hCoV-19/Hong Kong/VM20035710/2020	EPI_ISL_1963589
hCoV-19/Hong Kong/VM21006178/2021	EPI_ISL_1963590
hCoV-19/Hong Kong/VM21006467/2021	EPI_ISL_1963591
hCoV-19/Hong Kong/VM21008310/2021	EPI_ISL_1963592
hCoV-19/Hong Kong/VM21009395/2021	EPI_ISL_1963593
hCoV-19/Hong Kong/CM21000141/2021	EPI_ISL_1963594
hCoV-19/Hong Kong/VM21009999/2021	EPI_ISL_1963595
hCoV-19/Hong Kong/VM21010780/2021	EPI_ISL_1963596
hCoV-19/Hong Kong/VM21010776/2021	EPI_ISL_1963597

Virus name	GISAID no.
hCoV-19/Hong Kong/VM21013677/2021	EPI_ISL_1963598
hCoV-19/Hong Kong/VM21013785/2021	EPI_ISL_1963599
hCoV-19/Hong Kong/CM21000184/2021	EPI_ISL_1963600
hCoV-19/Hong Kong/VQ21052023/2021	EPI_ISL_1963601
hCoV-19/Hong Kong/VQ21051174/2021	EPI_ISL_1963602
hCoV-19/Hong Kong/CM21000177/2021	EPI_ISL_1963603
hCoV-19/Hong Kong/VM21013309/2021	EPI_ISL_1963604
hCoV-19/Hong Kong/VM21013535/2021	EPI_ISL_1963605
hCoV-19/Hong Kong/CM21000171/2021	EPI_ISL_1963606
hCoV-19/Hong Kong/CM21000169/2021	EPI_ISL_1963607
hCoV-19/Hong Kong/VM21012351/2021	EPI_ISL_1963608
hCoV-19/Hong Kong/VM21013848/2021	EPI_ISL_1963609
hCoV-19/Hong Kong/VM21013846/2021	EPI_ISL_1963610
hCoV-19/Hong Kong/CH21027623/2021	EPI_ISL_1963611
hCoV-19/Hong Kong/CM21000191/2021	EPI_ISL_1963612
hCoV-19/Hong Kong/CM21000193/2021	EPI_ISL_1963613
hCoV-19/Hong Kong/VM21014692/2021	EPI_ISL_1963614
hCoV-19/Hong Kong/VM21014363/2021	EPI_ISL_1963615
hCoV-19/Hong Kong/VM21014699/2021	EPI_ISL_1963616
hCoV-19/Hong Kong/CM21000195/2021	EPI_ISL_1963617
hCoV-19/Hong Kong/VM21015101/2021	EPI_ISL_1963618
hCoV-19/Hong Kong/VM21015013/2021	EPI_ISL_1963619
hCoV-19/Hong Kong/VM21015026/2021	EPI_ISL_1963620
hCoV-19/Hong Kong/VM21016277/2021	EPI_ISL_1963621
hCoV-19/Hong Kong/CM21000224/2021	EPI_ISL_1963622
hCoV-19/Hong Kong/CM21000226/2021	EPI_ISL_1963623
hCoV-19/Hong Kong/CM21000236/2021	EPI_ISL_1963624
hCoV-19/Hong Kong/CM21000238/2021	EPI_ISL_1963625
hCoV-19/Hong Kong/VM21015887/2021	EPI_ISL_1963626
hCoV-19/Hong Kong/VM21002742/2021	EPI_ISL_1963627
hCoV-19/Hong Kong/VM21003928/2021	EPI_ISL_1963628
hCoV-19/Hong Kong/VM21017523/2021	EPI_ISL_1963629
hCoV-19/Hong Kong/VM21017524/2021	EPI_ISL_1963630
hCoV-19/Hong Kong/VM21017055/2021	EPI_ISL_1963631
hCoV-19/Hong Kong/VM21017054/2021	EPI_ISL_1963632
hCoV-19/Hong Kong/VQ21059088/2021	EPI_ISL_1963633
hCoV-19/Hong Kong/CM21000251/2021	EPI_ISL_1963634
hCoV-19/Hong Kong/CM21000252/2021	EPI_ISL_1963635
hCoV-19/Hong Kong/VM21017238/2021	EPI_ISL_1963636
hCoV-19/Hong Kong/CM21000253/2021	EPI_ISL_1963637
hCoV-19/Hong Kong/VM21017519/2021	EPI_ISL_1963638
hCoV-19/Hong Kong/VM21017521/2021	EPI_ISL_1963639
hCoV-19/Hong Kong/VM21016396/2021	EPI_ISL_1963640
hCoV-19/Hong Kong/CM21000239/2021	EPI_ISL_1963641
hCoV-19/Hong Kong/CM21000248/2021	EPI_ISL_1963642
hCoV-19/Hong Kong/VM21016984/2021	EPI_ISL_1963643
hCoV-19/Hong Kong/VM21017052/2021	EPI_ISL_1963644

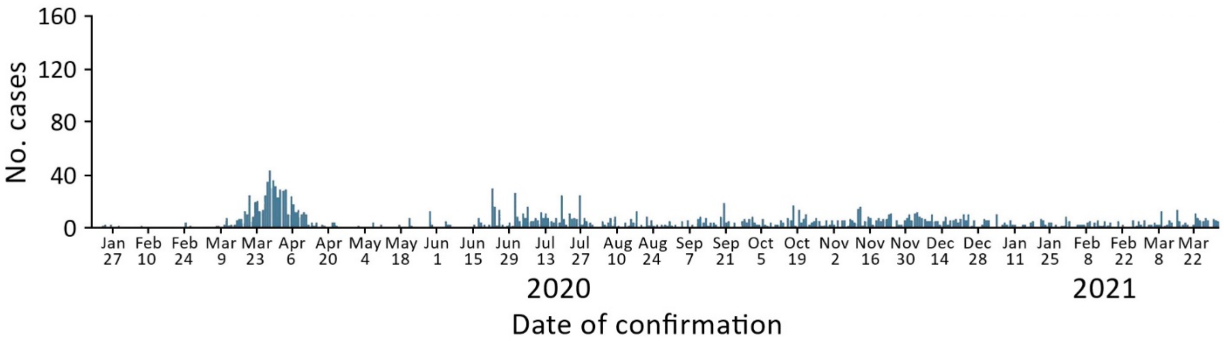

Appendix Figure. Daily number of COVID-19 cases imported by travellers to Hong Kong, January 2020–March 2021.
